# Multi-species transcriptome meta-analysis of the response to retinoic acid in vertebrates and comparative analysis of the effects of retinol and retinoic acid on gene expression in LMH cells

**DOI:** 10.1186/s12864-021-07451-2

**Published:** 2021-03-02

**Authors:** Clemens Falker-Gieske, Andrea Mott, Sören Franzenburg, Jens Tetens

**Affiliations:** 1 Department of Animal Sciences, Georg-August-University, Burckhardtweg 2, 37077 Göttingen, Germany; 2 Institute of Clinical Molecular Biology, Christian-Albrechts-University of Kiel, Rosalind-Franklin-Straße 12, 24105 Kiel, Germany; 3 Center for Integrated Breeding Research, Georg-August-University, Albrecht-Thaer-Weg 3, 37075 Göttingen, Germany

**Keywords:** Retinoids, Retinoic acid, Retinol, RNA-seq, Meta-analysis, Transcriptomics

## Abstract

**Background:**

Retinol (RO) and its active metabolite retinoic acid (RA) are major regulators of gene expression in vertebrates and influence various processes like organ development, cell differentiation, and immune response. To characterize a general transcriptomic response to RA-exposure in vertebrates, independent of species- and tissue-specific effects, four publicly available RNA-Seq datasets from *Homo sapiens*, *Mus musculus*, and *Xenopus laevis* were analyzed. To increase species and cell-type diversity we generated RNA-seq data with chicken hepatocellular carcinoma (LMH) cells. Additionally, we compared the response of LMH cells to RA and RO at different time points.

**Results:**

By conducting a transcriptome meta-analysis, we identified three retinoic acid response core clusters (RARCCs) consisting of 27 interacting proteins, seven of which have not been associated with retinoids yet. Comparison of the transcriptional response of LMH cells to RO and RA exposure at different time points led to the identification of non-coding RNAs (ncRNAs) that are only differentially expressed (DE) during the early response.

**Conclusions:**

We propose that these RARCCs stand on top of a common regulatory RA hierarchy among vertebrates. Based on the protein sets included in these clusters we were able to identify an RA-response cluster, a control center type cluster, and a cluster that directs cell proliferation. Concerning the comparison of the cellular response to RA and RO we conclude that ncRNAs play an underestimated role in retinoid-mediated gene regulation.

**Supplementary Information:**

The online version contains supplementary material available at 10.1186/s12864-021-07451-2.

## Background

RO and its derivative RA belong to the vitamin A group of compounds. Derivatives of RO, termed retinoids, are involved in cell proliferation, differentiation, cell adhesion, and apoptosis in different types of vertebrate tissues [1] and play an important role in immunity (reviewed in [2]), male and female reproduction, embryonic development, and barrier integrity (reviewed in [3]). Hence, an in-depth understanding of gene regulation by retinoids is essential to understand their involvement in processes that affect health and diseases. RA is thought to be the main mediator of these effects and is therefore the most studied fat-soluble vitamin [3]. RA binds to different nuclear receptors that regulate gene expression through the binding to certain canonical sequences termed retinoic acid response-elements (RAREs). RAREs are typically two direct repeats of the sequence motif PuG (G/T) TCA with a variable spacer of 0–8 bases length (DR0-DR8) or are inverted repeats with no spacer (IR0) [4–6]. In 2002 Balmer and Blomhoff compiled a list of over 500 genes that have been identified to be regulatory targets of RA in different species and categorized them in a hierarchical manner. They identified 27 direct targets and 105 genes that can be modulated by RA [7]. Since these results might be biased by individual assumptions, we intended to generate an unbiased set of core RA response genes independent of tissue or cell type, exposure time, and species. A direct comparison of the transcriptomic responses of different cell and tissue types from different species has not been conducted so far. Hence, we performed a meta-analysis of RNA-seq data sets from five different vertebrate tissues and cells from four different species treated with RA for different periods of time. This led to the discovery of 91 DE genes. We were able to identify three RA response core interaction clusters, comprising 27 proteins of which seven to our knowledge have not been linked to RA. We propose that these networks of proteins are species- and tissue-spanning and mark the starting point of tissue-dependent downstream gene regulation after RA-stimulation.

Little focus has been put on elucidating whether RA and RO differ in their effect on gene expression. The only study conducted so far that compared gene expression in response to RA and RO investigated the application of both compounds to human skin. By histological assessment and real-time quantitative PCR (qPCR) for 12 target genes, Kong et al. concluded that the response of skin to RA and RO is similar with RA being more potent in its effect on gene and protein expression [8]. We conducted an in-depth comparison of the transcriptomic responses to RA and RO in chicken LMH cells. We thereby confirmed that RA exerts a stronger effect on down-stream targets and found only a 76% overlap in differentially expressed (DE) genes between both treatments. Furthermore, we observed differences in the early response to RA, which indicates an involvement of ncRNAs in the RA response.

## Results

### Transcriptome and differential expression analyses

To gain insights into species and tissue-specific effects of RA, the transcriptomic responses of five different cell and tissue types from four different species were compared: (i) LMH cells exposed to 100 nM RA for 4 h (*N* = 3, this study), (ii) human neuroblastoma cells (SH-SY5Y) exposed to 1 μM RA for 24 h (*N* = 2, BioProject PRJEB6636) [9], (iii) murine embryonic stem cells (mESCs) exposed to 1 μM RA for 48 h (*N* = 3, BioProject PRJNA274740) [10], (iv) murine lymphoblasts (mLympho) exposed to 1 μM of RA for 2 h (*N* = 4, BioProject PRJNA282594) [11], and in vitro-generated pancreatic explants from *Xenopus laevis* (Xenopus) exposed to 5 μM RA for 1 h (*N* = 2, each sample contained ~ 50 pooled explants, BioProject PRJNA448780) [12]. Additionally, we performed a comparative analysis of the response of LMH cells to RA and RO after 1 h and 4 h treatments. Alignment metrics after mapping of RNAseq reads with TopHat are shown in Table 1 and detailed results per sample and dataset are summarized in Additional file 1.

**Table 1:** Summary statistics of transcriptome mappings of all datasets used in the study

Dataset	Instrument	Read length	Avg. no. of reads	SD no. of reads	Aligned reads (%)	Multiple alingments (%)	Exon coverage	BioProject	Reference
LMH cells	Illumina NovaSeq	2 × 50 bp	56,194,679	7,049,990	92.2	2.4	51.3x	PRJNA667585	This study
SH-SY5Y cells	Illumina Genome Analyzer IIx	1 × 35 bp	20,527,389	6,226,040	99.1	32.8	4x	PRJEB6636	[9]
mESCs	Illumina HiSeq 2000	1 × 50 bp	31,145,345	12,850,360	97.4	18.1	9.1x	PRJNA274740	[10]
mLympho	Illumina HiSeq 2500	2 × 100 bp	45,280,435	16,575,120	92.2	8.1	52.7x	PRJNA282594	[11]
Xenopus	Illumina HiSeq 2000	1 × 50 bp	23,289,030	1,851,281	94.5	6.5	11.5x	PRJNA448780	[12]

### A meta-analysis of the effects of retinoic acid on gene expression in different vertebrate tissues

**Fig. 1:**
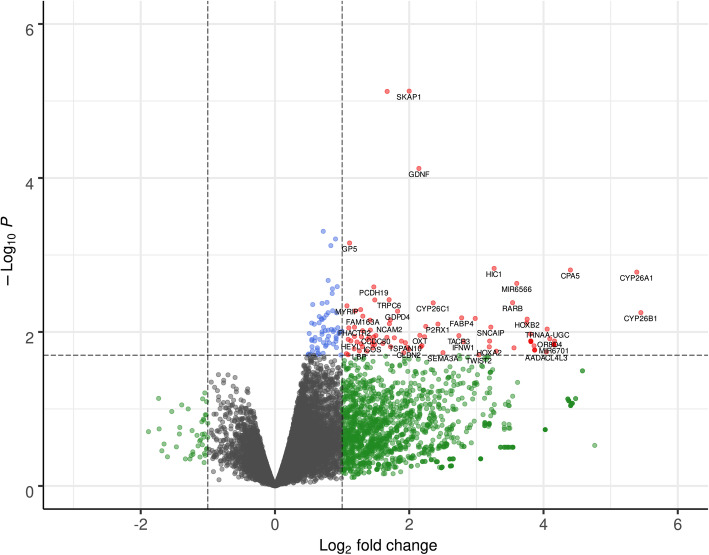
Volcano plot of differentially expressed genes from a transcriptome meta-analysis that was conducted with MetaVolcanoR. The results of each respective differential expression analysis from chicken hepatocellular carcinoma (LMH) cells, human neuroblastoma cells (SHSY5Y), murine embryonic stem cells (mESCs), murine lymphoblasts (mLympho), and in vitro-generated pancreatic explants from *Xenopus laevis* (Xenopus) after exposure to retinoic acid were used as input data. Red dots represent transcripts with a *p*-value < 0.02 and a LFC > 1

The results of all DE analyses are summarized in Additional file 2. DE analysis of the datasets by comparing untreated with RA treated cells or tissues led to the discovery of 139 DE genes in LMH cells (73.4% upregulated), 164 DE genes in SH-SY5Y cells (68.9% upregulated), 3967 DE genes in mESCs (56.8% upregulated), 679 DE genes in murine lymphocytes (57.4% upregulated), and 48 DE genes in Xenopus (97.9% upregulated; p-adj < 0.01, abs. LFC > 1). Concordance of DE genes between the five analyses is represented by a Venn diagram (Additional file 3) and summarized in Additional file 4. None of the discovered DE genes were common in all five systems and the majority of DE genes were limited to each respective cell/tissue type. An overlap in at least two systems could be observed for 262 out of all DE genes. Due to the little overlap between the five datasets, we conducted a meta-analysis with MetaVolcanoR. This led to the discovery of 91 DE genes with a *p*-value < 0.02 and abs. LFC > 1 (Fig. 1; complete results are summarized in Additional file 2), all of which were upregulated. The 20 highest ranked DE genes are shown in Table 2. Four transcription factors could be detected among DE genes with the PANTHER classification system [13]: *HEYL* (LFC = 1.130, *p*-value = 1.31 × 10^− 2^), *HIC1* (LFC = 3.264, *p*-value = 1.49 × 10^− 3^), *RARB* (LFC = 3.539, *p*-value = 4.17 × 4^− 3^), and *TWIST2* (LFC = 3.037, *p*-value = 1.99 × 10^− 2^).

**Table 2:** Top 20 DE genes from a multi-species transcriptome meta-analysis. RNA-seq data from five different cell types from four different vertebrate species after retinoic acid exposure were subjected to differential expression analysis and used as input for a meta-analysis

Symbol	LFC	*p*-value
SKAP1	1.999	7.44 × 10^−6^
SLCO2B1	1.671	7.51 × 10^− 6^
GDNF	2.144	7.52 × 10^− 5^
SMAD3	1.111	4.93 × 10^− 4^
GP5	3.264	6.99 × 10^− 4^
HIVEP2	4.400	7.56 × 10^− 4^
HIC1	5.389	1.49 × 10^− 3^
CYP26A1	3.601	1.67 × 10^− 3^
MIR6566	1.472	2.34 × 10^− 3^
ETS2	1.700	2.75 × 10^− 3^
TDRD9	1.487	3.16 × 10^− 3^
ERMN	3.539	3.84 × 10^− 3^
COL24A1	2.356	4.13 × 10^− 3^
TTYH3	1.072	4.25 × 10^− 3^
NOTCH2	1.276	4.40 × 10^− 3^
GPR61	1.196	5.13 × 10^− 3^
ADAM28	1.826	5.30 × 10^− 3^
KCNIP1	5.450	5.33 × 10^− 3^
NOXA1	1.309	5.65 × 10^− 3^
STXBP4	2.781	6.31 × 10^− 3^

*ADAM28* Disintegrin and metalloproteinase domain-containing protein 28, *COL24A1* Collagen alpha-1(XXIV) chain, *CYP26A1* Cytochrome P450 26A1, *ERMN* Ermin, *ETS2* Protein C-ets-2, *GDNF* Glial cell line-derived neurotrophic factor, *GP5* Platelet glycoprotein V, *GPR61* G-protein coupled receptor 61, *HIC1* Hypermethylated in cancer 1 protein, *HIVEP2* Transcription factor HIVEP2, *KCNIP1* Kv channel-interacting protein 1, *MIR6566* MicroRNA 6566, *NOTCH2* Neurogenic locus notch homolog protein 2, *NOXA1* NADPH oxidase activator 1, *SKAP1* Src kinase-associated phosphoprotein 1, *SLCO2B1* Solute carrier organic anion transporter family member 2B1, *SMAD3* Mothers against decapentaplegic homolog 3, *STXBP4* Syntaxin-binding protein 4, *TDRD9* ATP-dependent RNA helicase TDRD9, *TTYH3* Protein tweety homolog 3

**Fig. 2:**
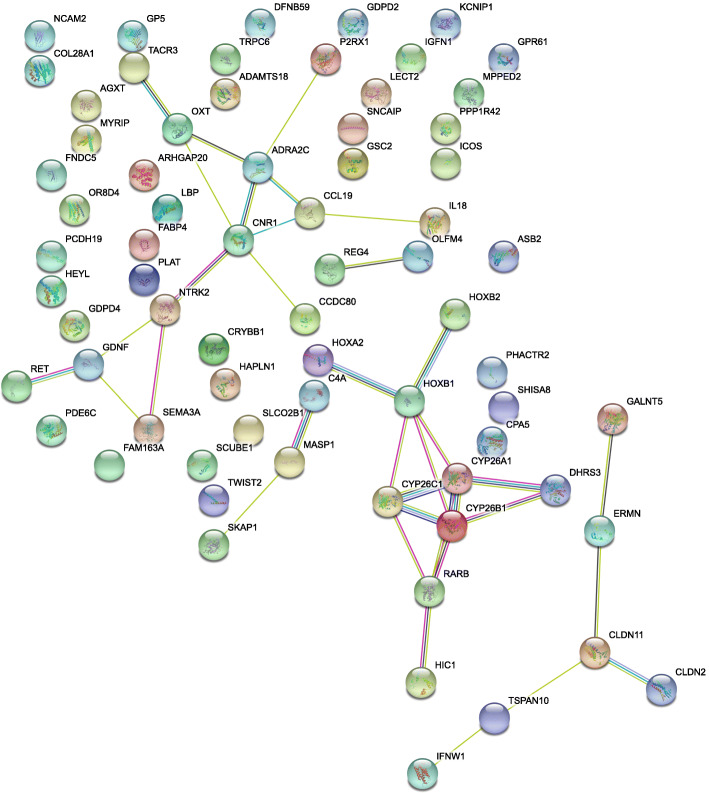
Protein interaction analysis of differentially expressed genes from a transcriptome meta-analysis that was conducted with differential expression data from chicken hepatocellular carcinoma cells, human neuroblastoma cells, murine embryonic stem cells, murine lymphoblasts, and in vitro-generated pancreatic explants from *Xenopus laevis* after exposure to retinoic acid . DE genes with *p*-values < 0.02 and LFC > 1 were used for the analysis

**Fig. 3:**
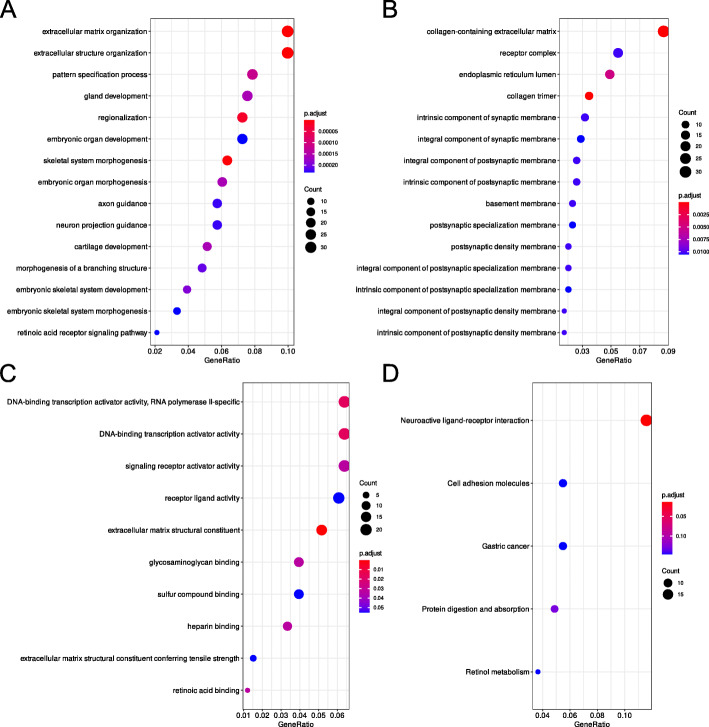
Gene cluster analysis of differentially expressed genes from a transcriptome meta-analysis that was conducted with differential expression data from chicken hepatocellular carcinoma cells, human neuroblastoma cells, murine embryonic stem cells, murine lymphoblasts, and in vitro-generated pancreatic explants from *Xenopus laevis* after exposure to retinoic acid. DE genes with a *p*-value < 0.05 and an abs. LFC > 0.5 were used for the analysis. **a** GO biological processes, **b** GO cellular components, **c** GO molecular functions, and **d** KEGG pathways

To identify potential functional protein clusters among DE gene from the meta-analysis we performed protein interaction network analysis with STRING. The analysis revealed significantly more interactions than expected (Fig. 2, number of edges: 36, expected number of edges: 13, PPI enrichment *p*-value: 2.2 × 10^− 7^). Three distinct interaction clusters were identified: Cluster (i) contains the proteins ADRA2C, CCDC80, CCL19, CNR1, GDNF, IL18, NTRK2, OXT, P2RX1, RET, SEMA3A, and TACR3, cluster (ii) consists of the proteins CYP26A1, CYP26B1, CYP26C1, DHRS3, HIC1, HOXA2, HOXB1, HOXB2, and RARB and cluster (iii) contains CLDN11, CLDN2, ERMN, GALNT5, IFNW1, and TSPAN10. To identify general functions of RA, which are common among the five analyzed datasets we performed a gene cluster analysis with clusterProfiler using DE genes with *p*-values < 0.05 and abs. LFC > 0.5 as input data. Results are shown in Fig. 3 (complete analysis output is summarized in Additional file 5). GO biological processes affected by DE genes from the meta-analysis (Fig. 3a) are mainly involved in morphogenesis, development, and extracellular organization as well as “axon guidance” and “neuron projection guidance”. In regard to GO cellular components (Fig. 3b), most of the terms involve synaptic and postsynaptic membranes. The term with the lowest *p*-value and highest GeneRatio is “collagen-containing extracellular matrix”. GO molecular functions, which are enriched in the meta-analysis (Fig. 3c) involve transcription activator activity, receptor activity, extracellular matrix structure, and binding of sulfur, heparin, and retinoic acid. In regard to KEGG pathways (Fig. 3d) only “Neuroactive ligand-receptor interaction” reached statistical significance (*p*-value < 0.05).

### Comparison of early and late RA and RO response in LMH cells

**Fig. 4:**
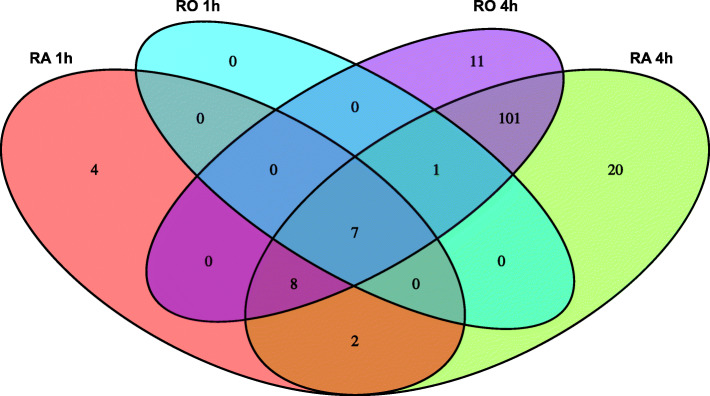
Venn diagram of differentially expressed genes in LMH cells after exposure to retinoic acid for 1 h (RA_1h), retinoic acid for 4 h (RA_4h), retinol for 1 h (RO_1h), and retinol for 4 h (RO_4h)

**Fig. 5:**
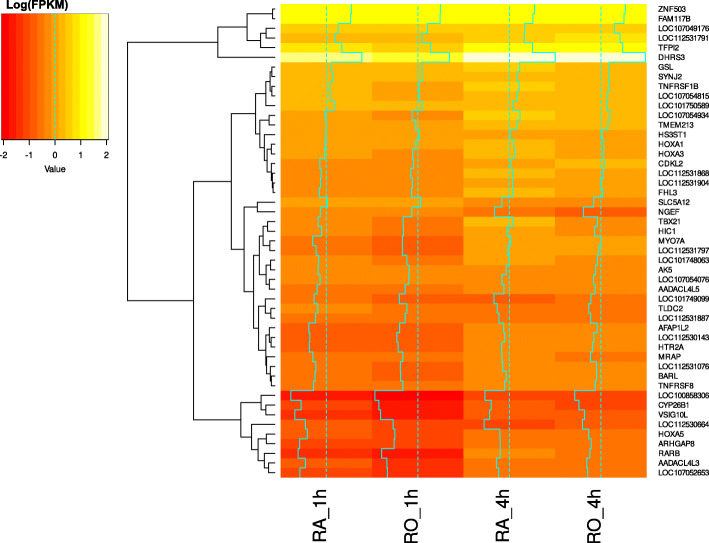
Heatmap of DE genes that differ between retinoic acid and retinol treatment in LMH cells: Log(FPKM) values of genes with at least 1.2-fold difference in FPKM values between retinoic acid and retinol treatment after 1 h or 4 h hours are shown. Cells treated with retinoic acid for 1 h (RA_1h), were compared with cell treated with retinol for 1 h (RO_1h) and cells treated with retinoic acid for 4 h (RA_4h), were compared with cell treated with retinol for 4 h (RO_4h)

To compare the response of hepatic cells to RA and RO we analyzed differential expression in LMH cells treated with RA and RO for time periods of 1 h and 4 h. This led to the discovery of 21 DE genes after 1 h of RA treatment, 139 DE genes after 4 h of RA treatment, 8 DE genes after 1 h of RO treatment, and 128 DE genes after 4 h of RO treatment (p-adj < 0.01, abs. LFC > 1). The majority of DE genes were upregulated (95% RA 1 h, 76% RA 4 h, 100% RO 1 h, 75% RO 4 h). Volcano plots of DE genes after RA and RO exposure for both time points are shown in Additional file 6 and the complete results of the DE analysis are summarized in Additional file 2. The numbers of common and discordant DE genes from all four treatments are summarized in a Venn diagram (Fig. 4, complete results Additional file 7). Only seven genes were commonly DE in all four treatments: *AADACL4L5*, *BARL*, *CYP8B1*, *LEKR1*, *LOC107054076* (ncRNA), *RBPMS*, *TBX21*, and *TNFRSF8*. The genes *ATF3*, *BAIAP2*, *LOC101749099* (ncRNA), and *LOC101750589* (ncRNA) are exclusive for the early response to RA. RO specific genes are *ADAMTS9*, *AFAP1L2*, *ARHGAP24*, *CDKL2*, *LOC112530664* (ncRNA), *LOC112531076* (pseudo-gene), *LOC112531755* (ncRNA), *LOC112531791* (ncRNA), *PALMD*, *RUNX1T1*, and *VSIG10L*. A total number of 26 genes were DE in a RA-dependent manner whereas a major overlap of 101 DE genes between RA and RO treatment after 4 h of exposure was observed. Genes with differences in expression between RA and RO treatment (min. 1.2-fold difference in Fragments per kilobase of exon model per million reads mapped (FPKM) values) after 1 h or 4 h are depicted in a heatmap (Fig. 5). The majority of differences in FPKM values were found between the time points, which were not considered in the heatmap. The genes with the highest differences in FPKM values between RA and RO treatment are listed in Table 3. The most distinct genes between both treatments are *AADACL4L3*, *CYP26B1*, *HIC1*, and *RARB*, all of which differ most in the early response and show stronger upregulation after RA stimulation. The only genes with a stronger response to RO (FPKM fold-difference > 1.2) are *ARHGAP8*, *CDKL2*, *HS3ST1*, and *SLC5A12* after 1 h of exposure as well as *AFAP1L2*, *LOC101749099* (ncRNA), *LOC112530664* (ncRNA), *LOC112531076* (pseudo-gene), *LOC112531791* (ncRNA), and *VSIG10L* after 4 h of exposure. Among the genes with the highest differences in FPKM values between RA and RO treatment are seven ncRNAs.

**Table 3:** Genes that are differentially expressed in at least one treatment with a > 1.5 fold difference in FPKM values between retinoic acid and retinol treatment in LMH cells. Cells treated with retinoic acid for 1 h (RA_1h), were compared with cell treated with retinol for 1 h (RO_1h), and cells treated with retinoic acid for 4 h (RA_4h), were compared with cell treated with retinol for 4 h (RO_4h)

Symbol	FPKM(RA_1h)	FPKM(RA_4h)	FPKM(RO_1h)	FPKM(RO_4h)	max(1 h)/min(1 h)	max(4 h)/min(4 h)
*AADACL4L3*	0.110	0.260	0.039	0.228	2.800	1.140
*BARL*	0.310	0.708	0.165	0.626	1.874	1.131
*CYP26B1*	0.072	0.152	0.017	0.102	4.223	1.491
*HIC1*	0.599	0.810	0.219	0.514	2.728	1.575
*HOXA5*	0.137	0.369	0.091	0.280	1.507	1.318
*LOC100858306*^*a*^	0.025	0.077	0.011	0.066	2.170	1.169
*LOC101749099*^*a*^	0.277	0.199	0.147	0.357	1.885	1.796
*LOC107052653*^*a*^	0.062	0.328	0.041	0.256	1.503	1.279
*LOC107054934*^*a*^	0.878	3.019	0.554	2.576	1.586	1.172
*LOC112530143*^*a*^	0.176	0.613	0.112	0.489	1.568	1.253
*LOC112530664*^*a*^	0.113	0.072	0.077	0.149	1.467	2.063
*LOC112531076*	0.334	0.492	0.160	0.623	2.093	1.266
*LOC112531791*^*a*^	2.526	4.686	1.514	7.300	1.669	1.558
*LOC112531797*	0.337	0.856	0.199	0.799	1.699	1.071
*RARB*	0.053	0.387	0.023	0.324	2.305	1.195
*TBX21*	0.426	1.418	0.216	0.694	1.970	2.042
*VSIG10L*	0.038	0.127	0.021	0.181	1.825	1.422

*AADACL4L3* arylacetamide deacetylase like 3C, *BARL* bile acid receptor-like, *CYP26B1* Cytochrome P450 26B1, *HIC1* Hypermethylated in cancer 1 protein, *HOXA5* Homeobox protein Hox-A5, *LOC100858306* ncRNA, *LOC101749099* ncRNA, *LOC107052653* ncRNA, *LOC107054934* ncRNA, *LOC112530143* ncRNA, *LOC112530664* ncRNA, *LOC112531076* zinc finger protein 664-like, *LOC112531791* ncRNA, *LOC112531797* atherin-like, *RARB* Retinoic acid receptor beta, *TBX21* T-box transcription factor TBX21, *VSIG10L* V-set and immunoglobulin domain-containing protein 10-like

^a^ncRNAs

To elucidate if the DE genes that we identified by exposing LMH cells to RA and RO might be RARE-regulated the chicken reference genome (GCF_000002315.5) was screened for RAREs (DR0-DR8 and IR0). The numbers of RAREs in the vicinity of DE genes (up to 10 kb upstream of transcript start and 10 kb downstream of transcript end) are summarized in Additional file 8. We detected RAREs in the vicinity of 103 out of 150 DE genes from all four treatments with an average of 2.07 RAREs per gene. The average occurrence of RAREs per gene in the genome is 0.77. Genes with ten or more RAREs close to the gene coding region are *ARHGAP24*, *OBSCN*, *RARB*, *STARD13*, and *TOX*.

**Fig. 6:**
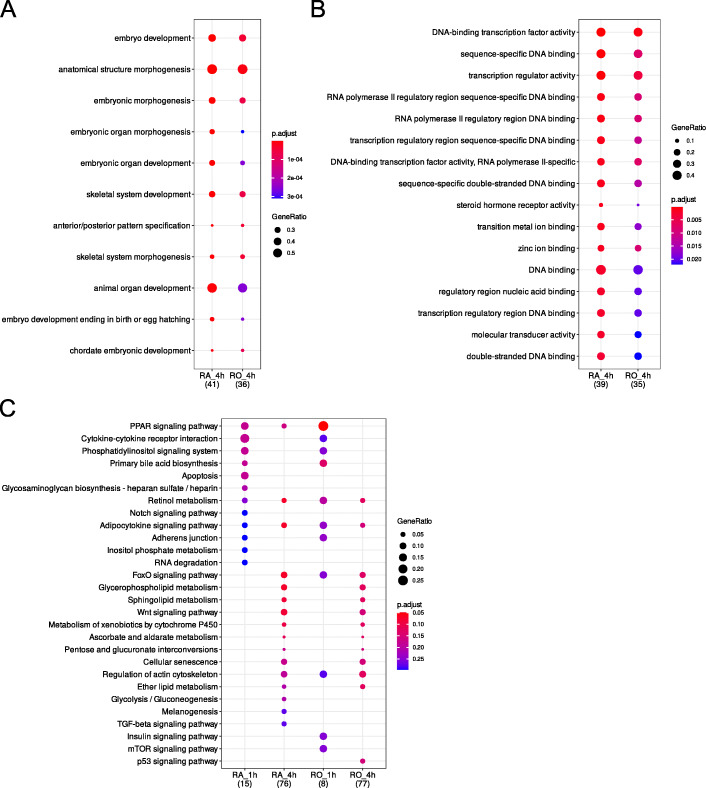
Gene cluster comparison of differentially expressed genes in LMH cells with clusterProfiler after exposure to retinoic acid for 1 h (RA_1h), retinoic acid for 4 h (RA_4h), retinol for 1 h (RO_1h), and retinol for 4 h (RO_4h). Sufficient differentially expressed genes were found to analyze **a** GO biological processes, **b** GO molecular functions, and **c** KEGG pathways

To find out whether certain protein interaction networks are differentially affected by RA and RO treatment the products of DE genes after 4 h of exposure to RA and RO were subjected to protein interaction network analyses with STRING [14] (interaction graphs in Additional file 9). In both cases, the networks had significantly more interactions than expected (RA treatment: number of edges: 41, expected number of edges: 21. PPI enrichment *p*-value: 7.29 × 10^− 5^; RO treatment: number of edges: 28, expected number of edges: 17. PPI enrichment *p*-value: 0.0107). With a higher level of significance and a higher number of edges, we could observe a higher degree of protein interaction among RA-regulated genes. Among those genes is a cluster of HOX genes (*HOXA1*, *HOXA3*, *HOXA5*, *HOXB3*, and *HOXB4*) and a cluster of genes primarily involved in bone development (*MSX2*, *RUNX2*, *THBS1*, *TNFRSF11B*, *TOR4A*). The interaction cluster surrounding *RARB* is larger (15 proteins) in RA-treated cells compared to RO-treated cells (8 genes). One interaction cluster that both treatments have in common consists of four genes encoding proteins with G protein-coupled receptor activity: *BDKRB2*, *GPR37L1*, *GRM8*, and *HTR2A*. To investigate if short- and long-term RA and RO exposure have different effects on the cellular response we performed a cluster analysis of DE genes (p-adj < 0.01, abs. LCF > 0.5) with clusterProfiler (complete analysis output is summarized in Additional file 5). The analysis revealed that treatment with RA and RO leads to an increase in GO biological processes associated with embryo, organ and skeletal system development and morphogenesis. RA acts more potent on the GO terms “embryo organ morphogenesis”, “embryonic organ development”, “animal organ development”, and “embryo development ending in birth or egg hatching” (Fig. 6a). The impact of RA on GO molecular functions was significantly higher as compared to RO with the majority of GO terms related to transcription, DNA-binding, gene expression, and metal ion binding. Comparable *p*-values between cells treated with RA and RO were only found for the GO terms “DNA-binding transcription factor activity” and “transcription regulator activity” (Fig. 6b). Due to the limited amount of DE genes detected for the 1 h time point comparison of early and late response to RA and RO was only possible in the KEGG pathway analysis. KEGG pathways limited to the early response to RA and RO stimulation are “Cytokine-cytokine receptor interaction”, “Phosphatidylinositol signal system”, and “Primary bile acid biosynthesis”. “Apoptosis”, and “Glycosaminoglycan biosynthesis – heparin sulfate / heparin” were only affected after 1 h of RA stimulation and “Insulin signaling pathway” and “mTOR signaling pathway” after 1 h of RO exposure. An exposure of 4 h to RA and RO led to lower *p*-values in “Retinol metabolism” and “Adipocytokine signaling pathway”. Prominent effects of RA and RO limited to an exposure of 4 h include KEGG pathways related to lipid metabolism, “FoxO signaling pathway”, and “Wnt signaling pathway” (Fig. 6c).

## Discussion

### A meta-analysis of the transcriptomic responses to retinoic acid from different species

To gain further insights into RA-dependent gene-regulation we acquired four RNA-seq datasets from the NCBI SRA and mapped them to the most recent genome assembly of each respective species (*Homo sapiens*, *Mus musculus*, and *Xenopus laevis*). To increase species and cell type variety we performed RNA-seq on chicken hepatocellular carcinoma (LMH) cells after RA exposure. We ended up with whole transcriptome DE data from five different systems: chicken LMH cells, human neuroblastoma cell line SH-SY5Y, murine embryonic stem cells, murine lymphoblasts, and in vitro-generated pancreatic explants from *Xenopus laevis*. Data quality regarding read length and coverage was mixed. Exon coverages around 50x were achieved with LMH cells and murine lymphoblasts. Coverages around 10x for the mESC and Xenopus mappings are acceptable whereas a 4x coverage and a multiple alignment frequency of 32.8% in SH-SY5Y cells might have introduced bias into the DE analysis of this dataset. The high frequency of multiple alignments is a result of the short read length and the absence of paired reads. Hence, accuracy of the results may be affected by the relatively low to medium quality of the SH-SY5Y, mESC and Xenopus data sets. The number of DE genes in response to RA-stimulation appears to stand in direct relation to the transcriptional activity of the respective cell- and tissue-types. mESCs are by far most susceptible to RA-stimulation with almost 4000 DE genes, followed by murine lymphoblasts with 679 DE genes. However, the overlap of DE genes between the five systems was not very prominent (Additional file 3). Hence, we conducted a transcriptome meta-analysis with MetaVolcanoR. By using the random effect model we circumvent the introduction of bias by differing *p*-value dimensions between the five datasets. It produces summary LFCs based on the variance, which are then used to estimate summary *p*-values. This is followed by perturbation ranking with the topconfects approach, which ranks results by confident effect sizes. By combining RNA-seq data of five different cell types from four different species taken at different time points the experimental conditions are highly adverse. This enabled us to compile a high confidence RA-response gene set in this meta-analysis. We discovered 91 DE genes (Fig. 1) of which 27 are part of three protein interaction clusters (Fig. 2), which we term retinoic acid response core clusters (RARCCs). Of those 27 RARCC genes, seven have not been previously associated with RA: *P2RX1*, *TACR3*, *HIC1*, *ERMN*, *GALNT5*, *IFNW1*, and *TSPAN10* (Table 4).

**Table 4:** Summary of published data on genes that belong to the retinoic acid response protein interaction clusters

Gene	Protein	Cluster	Published data on RA-response
*ADRA2C*	Alpha-2C adrenergic receptor	i	Putative RXRβ and RARα binding motifs in the regulatory region [15]
*CCDC80*	Coiled-coil domain-containing protein 80	i	Upregulation in BM cells [16]
*CCL19*	C-C motif chemokine 19	i	Has been linked indirectly in numerous studies
*CNR1*	Cannabinoid receptor 1	i	Increase in hepatic expression [17]
*GDNF*	Glial cell line-derived neurotrophic factor	i	Downregulation in embryonic chicken sympathetic neurons [18]
*IL18*	Interleukin-18	i	Pathogen clearance in the gut via IL-18 [19]
*NTRK2*	BDNF/NT-3 growth factors receptor	i	Linked to RA-induced neuroblastoma differentiation [20]
*OXT*	Oxytocin-neurophysin 1	i	Responds to RA via retinoic acid response element in the OXT promoter region [21]
*P2RX1*	P2X purinoceptor 1	i	/
*RET*	Proto-oncogene tyrosine-protein kinase receptor Ret	i	Induction of neurite extension [22]
*SEMA3A*	Semaphorin-3A	i	Upregulation in SH-SY5Y cells [23]
*TACR3*	Neuromedin-K receptor	i	/
*CYP26A1*	Cytochrome P450 26A1	ii	RA hydroxylase [24]
*CYP26B1*	Cytochrome P450 26B1	ii	“
*CYP26C1*	Cytochrome P450 26C1	ii	“
*DHRS3*	Short-chain dehydrogenase/reductase 3	ii	reduces all-trans-retinal or oxidizes all-trans-retinol [25]
*HIC1*	Hypermethylated in cancer 1 protein	ii	/
*HOXA2*	Homeobox protein Hox-A2	ii	Hox gene expression activated by RA (reviewed in (20))
*HOXB1*	Homeobox protein Hox-B1	ii	“
*HOXB2*	Homeobox protein Hox-B2	ii	“
*RARB*	Retinoic acid receptor beta	ii	RA receptor [26]
*CLDN11*	Claudin-11	iii	Upregulation in glioma stem-like cells [27]
*CLDN2*	Claudin-2	iii	Upregulation in keratinocytes [28]
*ERMN*	Ermin	iii	/
*GALNT5*	Polypeptide N-acetylgalactosaminyltransferase 5	iii	/
*IFNW1*	Interferon omega-1	iii	/
*TSPAN10*	Tetraspanin-10	iii	/

Cluster (ii) is obviously on top of the RA response hierarchy since it contains proteins that directly metabolize RA, like CYP26A1, CYP26B1, CYP26C1, and DHRS3, or are well known RA receptors (RARB) and direct downstream targets (Hox genes). Cluster (i) appears to be a control center that exerts cell-type-dependent downstream responses. We come to that conclusion because it is an adverse mix of well-described RA responses, like neuronal development, pathogen response, and transcription initiation. Cluster (iii) is of special interest since it contains four genes that to our knowledge have not been associated with RA before. Based on their reported functions (UniProt) we assume that this RARCC is responsible for the stimulating effect that RA has on cell proliferation. Since the RARCCs are species and tissue spanning as well as time-point independent, we propose that these interaction networks are conserved among vertebrates and tissue/cell types. This knowledge might have implications for future research since these 27 genes can be considered on top of an RA-response hierarchy that gets more specialized downstream depending on cell and tissue type as well as time. The fact that 20 out of these 27 RARCC genes have been previously associated with the response to RA underscores the reliability of the meta-analysis approach. Furthermore, it makes the 7 newly discovered genes likely candidates to be RA-regulated, which needs to be further validated experimentally. Gene cluster analysis confirms previous findings concerning the cellular response to RA exposure (Fig. 3). These include neuron-specific GO terms and KEGG pathways as well as terms involved in cell proliferation, transcription, receptor activity, and binding of certain chemical compounds. RA is able to induce neuron-like phenotypes in stem cells and plays a major role in the switch between cell proliferation and neuronal differentiation (Reviewed in [29, 30]). That this function of RA is present on a higher level in the RA-response hierarchy is confirmed by our data. As Tang and Gudas outlined in their review article [31] RA is able to induce or inhibit cell proliferation in many cell types depending on the studied system. With our gene cluster analysis we can confirm that this is mostly a cell type-independent function of RA. This also holds true for the effect of RA on the activation of gene transcription in general. Further information on the effects of RA on gene transcription can be found in the overview article by Amann et al. [32]. In comparison with our findings for each respective dataset, these findings further indicate that we compiled a set of higher-level genes that orchestrate the RA-response further downstream depending on cell and tissue type. Only four DE transcription factors were detected in the meta-analysis of which three were previously described to be RA-responsive: *HIC1* [33], *RARB* [26], and *TWIST2* [34]. The transcriptional repressor *HEYL*, which is involved in cardiac gene expression [35], has not been linked to RA-mediated gene regulation and should be considered in further studies focusing on retinoid signaling in cardiac development (reviewed in [36]). The fact that virtually no genes were significantly downregulated in the meta-analysis of these five datasets confirms the notion that RA is mainly a transcriptional activator and not a repressor. Transcriptional repression seems to be regulated in a cell type-dependent manner. We saw downregulation of transcripts among the five individual datasets ranging from 43.2 to 2.1% of all DE genes. One gene that caught our attention is *TOX*, which is downregulated in three of the datasets: LMH cells, murine lymphoblasts, and mESCs. *TOX* is a transcriptional regulator that has not been linked to RA-biology. Our data indicate that *TOX* is a RARE regulated gene. We discovered 10 RAREs in the vicinity (up to 10 kb up- and down-stream) to the *TOX* gene in the chicken genome (Supporting Information 8). *TOX* is a diagnostic marker for cutaneous T-cell lymphomas (CTCL) and is overexpressed in affected CD4+ cells [37] Retinoids have been successfully used in the therapy of CTLC for over 30 years [38] and are still being used in T-cell lymphoma therapy [39]. The results from our analysis suggest that the success of retinoid cancer therapy is directly connected to *TOX* downregulation by RA. Identification of genetic polymorphisms related to *TOX* in patients that are susceptible to retinoid therapy and determination of the *TOX* expression state in CD4+ cells could aid in the tailored development of CTLC combination-therapy.

### Comparison of the retinoic acid and retinol response in chicken LMH cells

Since most past studies focused on the effects of RA on gene expression we wanted to investigate if the response to its dietary form RO is different. In summary, we were able to confirm the conclusion by Kong et al. that RA is a more potent modulator of gene expression compared to RO [8]. After 1 h incubation time, twice as many genes were DE in the RA treatment (Additional file 6) although the concentration of RO was ten times higher. We explain that effect by a delay in the response to RO since it first has to be oxidized to RA via retinal, which takes place in the cytosol of hepatic cells [40] and in microsomes [41]. Furthermore, the RO treated cells might produce physiological amounts of RA, which are very likely lower than the applied RA concentration in the other experimental group. After 4 h a comparable number of genes were DE in RA and RO stimulated cells. With an overlap of 76% in DE genes, the transcriptional responses to the two chemicals are similar, but not identical. By real-time PCR Kong et al. identified six genes (*COL1A1*, *COL3A1*, *CRABP2*, *FLG*, *TGM1*, and *TGM3*) that respond to RA and RO stimulation in human skin, none of which were DE in LMH cells, which were derived from chicken liver carcinoma. Only four genes were limited to the early response to RA, two of which were ncRNAs. Among RO-specific genes three out of eleven were also ncRNAs. Furthermore, the majority of DE transcripts that showed the strongest difference between both treatments are ncRNAs (Table 3). We hypothesize that ncRNAs play an important role in early processes of RA mediated gene regulation and in RO metabolism, which has already been described in the context of RA-mediated *TGM2* gene regulation [42]. BLAST search revealed that the ncRNA *LOC112530664* has binding properties to *TMEM168*, a gene that promotes cell proliferation [43]. Furthermore, *LOC112531791* shows sequence homology to *ABCC8*, which is a regulator of ATP-sensitive K+ channels and insulin release [44]. *LOC112530664* shows stronger upregulation after RO treatment in comparison to RA and *LOC112531791* is DE in an RO-dependent manner. Hence, these ncRNAs might be involved in RO metabolism by acting on those genes. To clarify the involvement of *TMEM168* and *ABBC8* in the cellular response to retinol requires further validation on the protein level.

*Hox* genes are crucial to vertebrate embryogenesis and are known to get activated by signaling cascades initiated by RA (reviewed in [45]). Our results indicate that RA has a stronger influence on *Hox* gene expression in comparison to RO. An interaction cluster consisting of five HOX proteins (HOXA1, HOXA3, HOXA5, HOXB3, and HOXB4) was detected with STRING (Additional file 9), whereas RO only led to the activation of three Hox genes. We assume that the conversion of RO to RA leads to lower RA levels in the cell compared to the direct administration of RA. Hence, the Hox gene response is less prominent after RO treatment. Another protein interaction cluster that we found in the RA response consists of MSX2, RUNX2, THBS1, TNFRSF11B, and TOR4A, all of which are involved in development. Dickson et al. demonstrated that embryonic chicken calvaria responds differently to RA and RO administration [46]. We can indirectly confirm these results since we did not find the mentioned bone development protein interaction cluster after RO administration. Other studies, which only focused on the RA response, found a stimulating effect on osteoclasts [47] and an inhibitory effect on osteoblasts [48], which suggests that RA leads to bone degradation rather than bone formation (discussed in this review [49]). The protein interaction cluster surrounding RARB is almost twice as large after RA treatment in comparison to RO treatment. Since the three direct interaction partners of RARB, namely NRIP1, ALDH1A3, and CYP26B1 are DE after both treatments, we assume that higher RA bioavailability in the cells leads to a higher downstream effect on gene expression of RARB targets. For instance, the RAR coregulatory NRIP1 [50], which is a transcription factor that regulates lipid and glucose metabolism in the liver [51], exhibits a stronger downstream effect after RA treatment. Furthermore, ALDH1A3 and CYP26B1, both of which are involved in retinoid metabolism [52, 53], may have more substrate to process in the presence of RA compared to RO. The interaction cluster containing the proteins BDKRB2, GPR37L1, GRM8, and HTR2A is present in the interaction maps of both treatments. Hence, G protein-coupled receptor activity seems to be equally responsive to RA and RO but has so far only been described for RA as a possible activator of Non-canonical Wnt Signaling [54].

Gene cluster analysis also revealed that RA is the more potent activator of gene expression in comparison to RO. Concerning GO biological processes (Fig. 6a) RA has a higher impact on terms that involve embryo and organ development a known function of RA as reviewed here [55]. The same holds true for terms belonging to GO molecular functions (Fig. 6b). Both compounds lead to an upregulation of terms affecting transcription, DNA-binding, gene expression, and metal ion binding with RA initiating a stronger response. KEGG pathway analysis (Fig. 6c) revealed some interesting insights into the early response of LMH cells to RA and RO. Pathways that are limited to 1 h of RA and RO treatment are “Cytokine-cytokine receptor interaction”, “Phosphatidylinositol signal system”, and “Primary bile acid biosynthesis”. An influence of RA on cytokines has been reported [56, 57] but not in a time-dependent manner. However, an immediate decrease in phosphatidylinositol turnover after RA exposure has been reported in neuroblastoma cells [58, 59]. Concerning the early response, we found differences in the response to RA and RO. Whereas early RA stimulation had an effect on “Apoptosis”, and “Glycosaminoglycan biosynthesis – heparin sulfate / heparin”, RO had an exclusive effect on “Insulin signaling pathway” and “mTOR signaling pathway”. With respect to insulin signaling, insulin was shown to regulate RA biosynthesis by upregulation of retinol dehydrogenase expression [60]. Our data suggest that vice versa, RO can upregulate genes that are involved in insulin metabolism in an immediate manner. Regarding mTOR signaling, synaptic RA receptors mediate hippocampal learning via mTOR dependent metaplasticity [61]. Since learning is an immediate and hippocampal consolidation a fast process [62], early activation of the mTOR signaling pathway after RO administration is conclusive.

Surprisingly, the response of LMH cells to RA and RO exposure with respect to KEGG “Retinol metabolism” genes was identical. After 1 h incubation time, only *DHRS3* was significantly upregulated in both treatments with a higher LFC after RA (2.455) exposure compared to RO (1.874). *DHRS3* reduces all-trans-retinal to all-trans-retinol or oxidizes all-trans-retinol to all-trans-retinal [25], most likely dependent on the stoichiometry between the two chemicals. Hence, lower expression in the presence of RO is conclusive, since it first has to be metabolized to RA via retinal as an intermediate product. After 4 h of RA and RO exposure, three additional genes were differentially expressed: *CYP26B1*, *RDH10*, and *UGT1A1*. *CYP26B1* hydroxylates RA to 4-OH-RA, 4-oxo-RA, or 18-OH-RA [52]. *RDH10*, which catalyzes the conversion of all-trans-retinol to all-trans-retinal [63], was downregulated after both treatments. *UGT1A1* activity leads to glucuronidation of RA [64], a detoxification process that takes place in the liver [65, 66]. Taken together upregulation of *CYP26B1*, and *UGT1A1* and downregulation of *RDH10* seem to be a detoxification mechanism to get rid of excess RA. If these genes play a role in retinoic acid syndrome [67] (reviewed in [68]) remains to be elucidated. The toxicity of RA in the treatment of acute promyelocytic leukemia has been described for the first time in a clinical case in 1992 [69]. We used a RA concentration of 100 nM and already observed a potentially toxic response. Hence, we conclude that a lower RA concentration or the application of RO in functional experiments might produce results that are closer to the natural response to retinoids. Furthermore, the studies discussed above utilized RA concentrations of 1 μM or higher, which may have introduced bias to the results by an overdose effect.

## Conclusions

By conduction a meta-analysis of the transcriptomic responses to RA exposure of five different vertebrate systems we were able to identify a core RA response gene set. From our results, we conclude that on a higher hierarchical level RA is an activator of transcription and that RA mediates transcription repression in a cell type-dependent manner. Furthermore, we conclude that RA exerts its downstream functions via three distinct protein interaction clusters: The largest cluster comprises diverse downstream targets of RA and might function as a control hub, which acts cell type-dependent. One mid-sized cluster almost exclusively contains well described direct RA targets, which we consider on top of a general gene expression hierarchy in vertebrates. The smallest cluster seems to be cell-type independent since it mainly contains genes, which are involved in cell proliferation – a general function of RA. The comparative analysis of the influence of RA and RO on gene expression in LMH cells confirmed that RA is a more potent inducer of gene expression. However, a discordance of 24% in DE genes caught our attention. Among those are two RA- and three RO-specific ncRNAs from which we conclude that ncRNAs play a central role in the early response to retinoids.

## Methods

### Cell culture

LMH cells were kindly provided by Prof. Schwemmle’s lab and cultured in MEM supplemented with 10% FBS. Culture conditions were 37 °C, 5% CO_2_ and 95% RH. 2 × 10^6^ Cells were seeded on a T25 flask and grown for 24 h. Cells were either treated with DMSO containing RA (final concentration 100 nM), Retinol (final concentration 1 μM) or DMSO only (control group) for 1 h or 4 h. The experiment was repeated three times independently.

### RNA isolation

Cells were washed with PBS, detached from the surface by adding 1 ml of accutase for 5 min, resuspended in twice the amount of medium, transferred to a 15 ml tube, centrifuged for 5 min at 400 g, resuspended in PBS, centrifuged for 5 min at 400 g, and the resulting cell pellet was used for RNA isolation. RNA was isolated with the RNeasy Mini Kit and QIAshredder columns (QIAGEN) according to the manual.

### NGS library preparation and sequencing

Procedures have already been described in a previous study [70]. Briefly: Total RNA input quality was evaluated on a TapeStation 4200 (Agilent, USA), and all samples showed a RIN score > 8. Samples were quantified with a fluorometric dye (Quant-IT, thermofisher, USA) and 500 ng per sample were used as input for the TruSeq stranded mRNA library kit (Illumina, USA) following the manufacturers manual. Resulting libraries showed a fragment size distribution of around 300 bp and were sequenced on a NovaSeq S2 Flowcell (Illumina, USA) with 50 bp paired-end reads.

### Resource datasets

The following sample comparisons were carried out in the differential expression analysis: LMH cells exposed to RA or RO were compared to DMSO-treated cells. All three treatments were applied for 1 h and 4 h. SH-SY5Y exposed to 1 μM RA for 24 h were compared to DMSO treated cells (BioProject PRJEB6636; RA-treated samples: ERR550444, ERR550446; DMSO Samples: ERR550449, ERR550450) [9]. mESCs exposed to 1 μM RA for 48 h were compared to untreated control cells (BioProject PRJNA274740; RA-treated samples: SRR1792530, SRR1792529, SRR1792531; control samples: SRR1792526, SRR1792528, SRR1792527) [10]. Murine lymphoblasts exposed to 1 μM of RA for 2 h were compared to DMSO treated cells (BioProject PRJNA282594; RA-treated samples: SRR2001796, SRR2001794, SRR2001797, SRR2001795; control samples: SRR2001790, SRR2001793, SRR2001791, SRR2001792) [11]. In vitro-generated pancreatic explants from *Xenopus laevis* (Xenopus) exposed to 5 μM RA for 1 h were compared to DMSO treated cells where each sample contained ~ 50 pooled explants (BioProject PRJNA448780; RA-treated samples: SRR6941647, SRR6941648; control samples: SRR6941648, SRR6941644) [12].

### Transcriptome analyses

DE analyses were carried out as previously described [70]. Briefly, quality control and trimming of raw sequencing reads was achieved with Trimmomatic version 0.36 (settings: PE -phred33 LEADING:3 TRAILING:3 SLIDINGWINDOW:4:15 MINLEN:36) [71]. Reads were aligned to the most recent reference genomes with TopHat version 2.1.0 (settings: --no-novel-juncs --min-isoform-fraction 0.0 --min-anchor-length 3 -r 192) [72]. Reference genomes (RefSeq assemblies) used for the alignment are *Gallus gallus* GCF_000002315.5, *Mus musculus* GCF_000001635.26, *Homo sapiens* GCF_000001405.39, and *Xenopus laevis* GCF_001663975.1. The R packages GenomicFeatures (Version 1.40.0) and summarizeOverlaps were used to count exon spanning reads [73]. DE analyses were conducted with DESeq2 (Version1.28.1) [74]. The R package EnhancedVolcano (Version 1.6.0) was used to generate volcano plots of DE results. Meta-analysis of DE datasets was carried out using the R package MetaVolcanoR (Version 1.2.0) using a random effect model. The following settings were applied: geneidcol = NULL, collaps = FALSE, cvar = FALSE, metathr = 0.01, ncores = 8. Gene Symbols of the input datasets were generalized by capitalizing all letters and removal of species specific pre- and suffixes.

### Functional analyses

Gene cluster comparison and visualization was achived with the R package clusterProfiler [75]. Gene symbols were converted to ensemble IDs with the clusterProfiler Biological Id Translator (bitr). GO term analyses were performed with enrichGO (settings: pAdjustMethod = “fdr”, pvalueCutoff = 1, qvalueCutoff = 0.25, readable = TRUE, minGSSize = 10). KEGG pathtway analysis was done with enrichKEGG (settings: pvalueCutoff = 1, pAdjustMethod = “BH”,minGSSize = 10, maxGSSize = 500, qvalueCutoff = 0.25, use_internal_data = FALSE). Plots were created with the dotplot function. Protein interaction maps were done with STRING (Version 11.0) [14] using default settings.

## Supplementary Information

**Additional file 1.** Complete alignment metrics after mapping with TopHat.

**Additional file 2.** Differential expression analyses results of all datasets after exposure to retinoic acid.

**Additional file 3.** Venn diagram of differentially expressed genes from all datasets after exposure to retinoic acid.

**Additional file 4.** Common differentially expressed genes among all datasets after exposure to retinoic acid.

**Additional file 5.** Results of clusterProfiler analyses of differentially expressed genes from the meta-analysis and LMH cells exposed to retinoic acid and retinol for 1 h and 4 h.

**Additional file 6.** Volcano plots of differentially expressed genes in LMH cells after exposure to retinoic acid and retinol for 1 h and 4 h.

**Additional file 7.** Common differentially expressed genes after exposure of LMH cells to retinoic acid and retinol for 1 h and 4 h.

**Additional file 8.** The numbers of RAREs in the vicinity of DE genes (up to 10 kb upstream of transcript start and 10 kb downstream of transcript end) in LMH cells after exposure to retinoic acid and retinol for 1 h and 4 h. LFCs for each gene and treatment are listed and results with a p-adj < 0.01 are indicated by *.

**Additional file 9.** Protein interaction network analysis results of genes that were differentially expressed in LMH cells after 4 h exposure to retinoic acid or retinol.

## Data Availability

The datasets with the following BioProject IDs were aquired from the NCBI SRA: PRJEB6636 (SH-SY5Y cells) https://trace.ncbi.nlm.nih.gov/Traces/sra/sra.cgi?study=ERP006185, PRJNA274740 (mESCs) https://trace.ncbi.nlm.nih.gov/Traces/sra/sra.cgi?study=SRP053290, PRJNA282594 (murine lymphoblasts) https://trace.ncbi.nlm.nih.gov/Traces/sra/sra.cgi?study=SRP057791, PRJNA448780 (*Xenopus laevis*) https://trace.ncbi.nlm.nih.gov/Traces/sra/sra.cgi?study=SRP137258 . The raw sequencing data that was generated for this study is accessible under BioProject ID PRJNA667585 (reviewer link: https://dataview.ncbi.nlm.nih.gov/object/PRJNA667585?reviewer=koal33cfclsaj7d2c7nifjipl2) and will be made publicly available upon publication. For the mapping of RNA-seq reads from LMH cells chicken genome version GCF_000002315.5 was used (genome assembly file: https://ftp.ncbi.nlm.nih.gov/genomes/all/GCF/000/002/315/GCF_000002315.5_GRCg6a/GCF_000002315.5_GRCg6a_genomic.fna.gz, genomic features file: https://ftp.ncbi.nlm.nih.gov/genomes/all/GCF/000/002/315/GCF_000002315.5_GRCg6a/GCF_000002315.5_GRCg6a_genomic.gff.gz). For the mapping of RNA-seq reads from murine cells *Mus musculus* genome version GCF_000002315.5 was used (genome assembly file: https://ftp.ncbi.nlm.nih.gov/genomes/all/GCF/000/001/635/GCF_000001635.26_GRCm38.p6/GCF_000001635.26_GRCm38.p6_genomic.fna.gz, genomic features file: https://ftp.ncbi.nlm.nih.gov/genomes/all/GCF/000/001/635/GCF_000001635.26_GRCm38.p6/GCF_000001635.26_GRCm38.p6_genomic.gff.gz). For the mapping of RNA-seq reads from SH-SY5Y cells *Homo sapiens* genome version GCF_000001405.39 was used (genome assembly file: https://ftp.ncbi.nlm.nih.gov/genomes/all/GCF/000/001/405/GCF_000001405.39_GRCh38.p13/GCF_000001405.39_GRCh38.p13_genomic.fna.gz, genomic features file: https://ftp.ncbi.nlm.nih.gov/genomes/all/GCF/000/001/405/GCF_000001405.39_GRCh38.p13/GCF_000001405.39_GRCh38.p13_genomic.gff.gz). For the mapping of RNA-seq reads from Xenopus tissue *Xenopus laevis* genome version GCF_001663975.1 was used (genome assembly file: https://ftp.ncbi.nlm.nih.gov/genomes/all/GCF/001/663/975/GCF_001663975.1_Xenopus_laevis_v2/GCF_001663975.1_Xenopus_laevis_v2_genomic.fna.gz, genomic features file: https://ftp.ncbi.nlm.nih.gov/genomes/all/GCF/001/663/975/GCF_001663975.1_Xenopus_laevis_v2/GCF_001663975.1_Xenopus_laevis_v2_genomic.gff.gz). For the mapping of RNA-seq reads from SH-SY5Y cells *Homo sapiens* genome version GCF_000001405.39 was used (genome assembly file: https://ftp.ncbi.nlm.nih.gov/genomes/all/GCF/000/001/405/GCF_000001405.39_GRCh38.p13/GCF_000001405.39_GRCh38.p13_genomic.fna.gz, genomic features file: https://ftp.ncbi.nlm.nih.gov/genomes/all/GCF/000/001/405/GCF_000001405.39_GRCh38.p13/GCF_000001405.39_GRCh38.p13_genomic.gff.gz).

## Abbreviations

CTCL: Cutaneous T-cell lymphoma
DE: Differentially expressed
DR: Direct repeat
FDR: False discovery rate
FPKM: Fragments per kilobase of exon model per million reads mapped
LFC: Log fold change
LMH cells: Chicken hepatocellular carcinoma cells
ncRNAs: Non-coding RNAs
p-adj: Adjusted *p*-value
qPCR: Real-time quantitative PCR
RA: Retinoic acid
RARCC: Retinoic acid response core cluster
RAREs: Retinoic acid response-elements
RNA-seq: RNA-sequencing
RO: Retinol
SRA: Sequence Read Archive
