# Supplementary material for: Multi-species transcriptome meta-analysis of the response to retinoic acid in vertebrates and comparative analysis of the effects of retinol and retinoic acid on gene expression in LMH cells
Source: BMC Genomics. 2021 Mar 2;22:146. doi: 10.1186/s12864-021-07451-2 (PMC7923837; doi:10.1186/s12864-021-07451-2)

Additional file 3: Venn diagram of differentially expressed genes in chicken hepatocellular carcinoma (LMH) cells, human neuroblastoma cells (SH-SY5Y), murine embryonic stem cells (mESCs), murine lymphoblasts (mLympho), and in vitro-generated pancreatic explants from Xenopus laevis (Xenopus) after exposure retinoic acid.

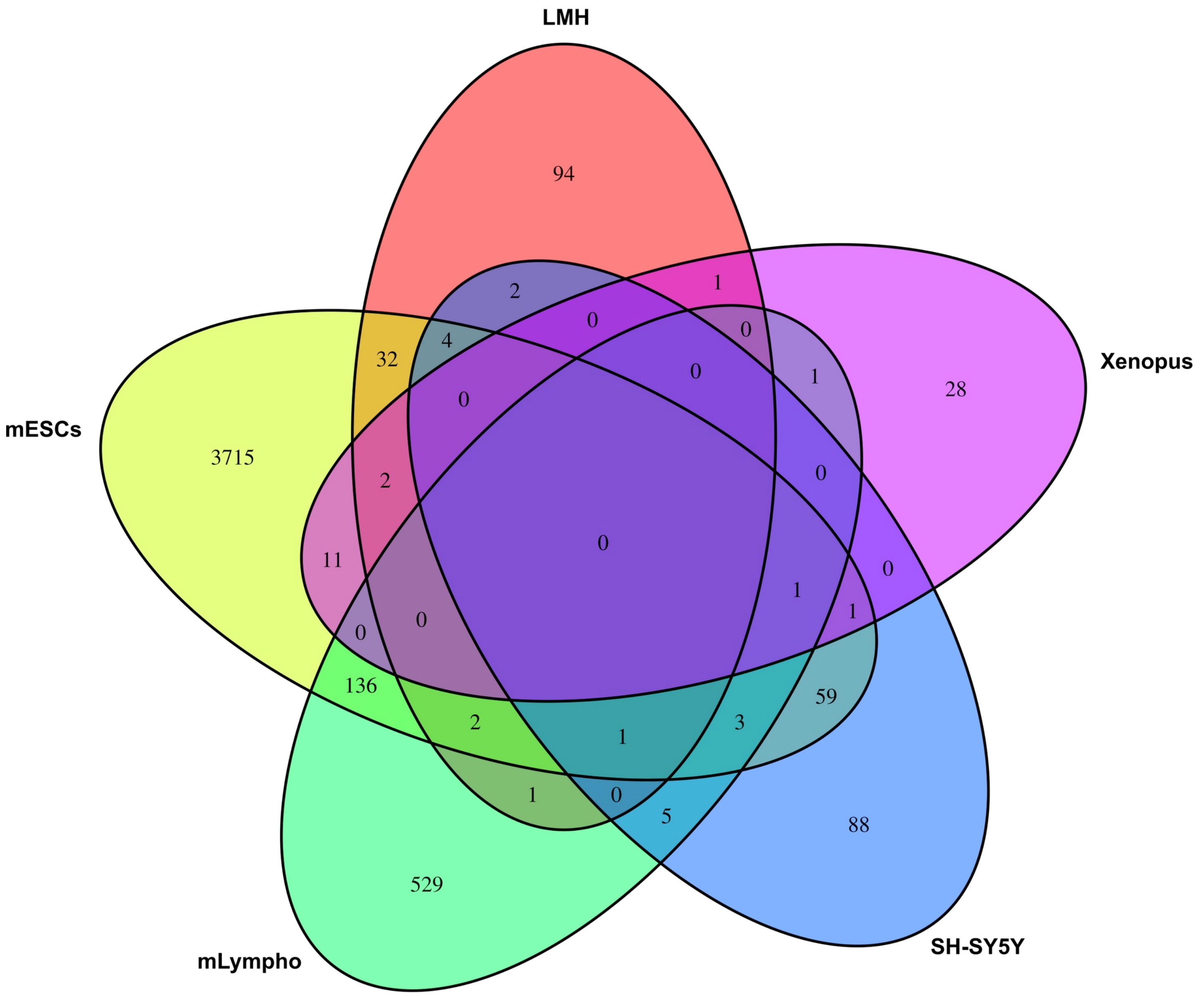

Supplement: Supplementary file 3 — Additional file 3. Venn diagram of differentially expressed genes from all datasets after exposure to retinoic acid. [file 12864_2021_7451_MOESM3_ESM.pdf]
