# Supplementary material for: Multi-species transcriptome meta-analysis of the response to retinoic acid in vertebrates and comparative analysis of the effects of retinol and retinoic acid on gene expression in LMH cells
Source: BMC Genomics. 2021 Mar 2;22:146. doi: 10.1186/s12864-021-07451-2 (PMC7923837; doi:10.1186/s12864-021-07451-2)

Additional file 6: Volcano plots of differentially expressed genes in LMH cells after exposure (A) to retinoic acid for 1h, (B) retinoic acid for 4h, (C) retinol for 1h, and (D) retinol for 4h. Red dots represent transcripts with a p-value < 0.00001 and a LFC > 1.

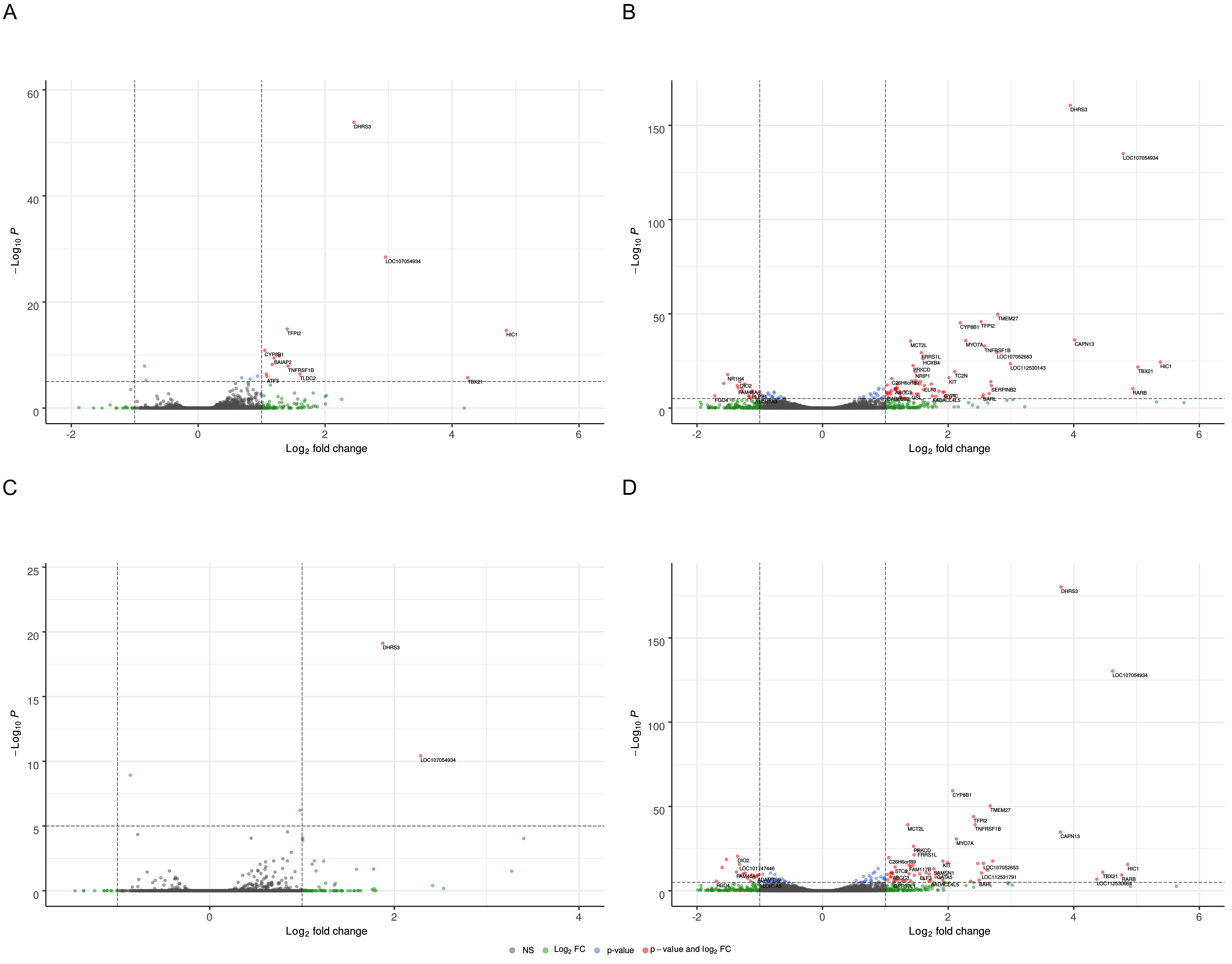

Supplement: Supplementary file 6 — Additional file 6. Volcano plots of differentially expressed genes in LMH cells after exposure to retinoic acid and retinol for 1 h and 4 h. [file 12864_2021_7451_MOESM6_ESM.pdf]
