# Supplementary material for: Multi-species transcriptome meta-analysis of the response to retinoic acid in vertebrates and comparative analysis of the effects of retinol and retinoic acid on gene expression in LMH cells
Source: BMC Genomics. 2021 Mar 2;22:146. doi: 10.1186/s12864-021-07451-2 (PMC7923837; doi:10.1186/s12864-021-07451-2)

Additional file 9A: Protein interaction network analysis of genes that were differentially expressed in LMH cells after 4h exposure to retinoic acid.

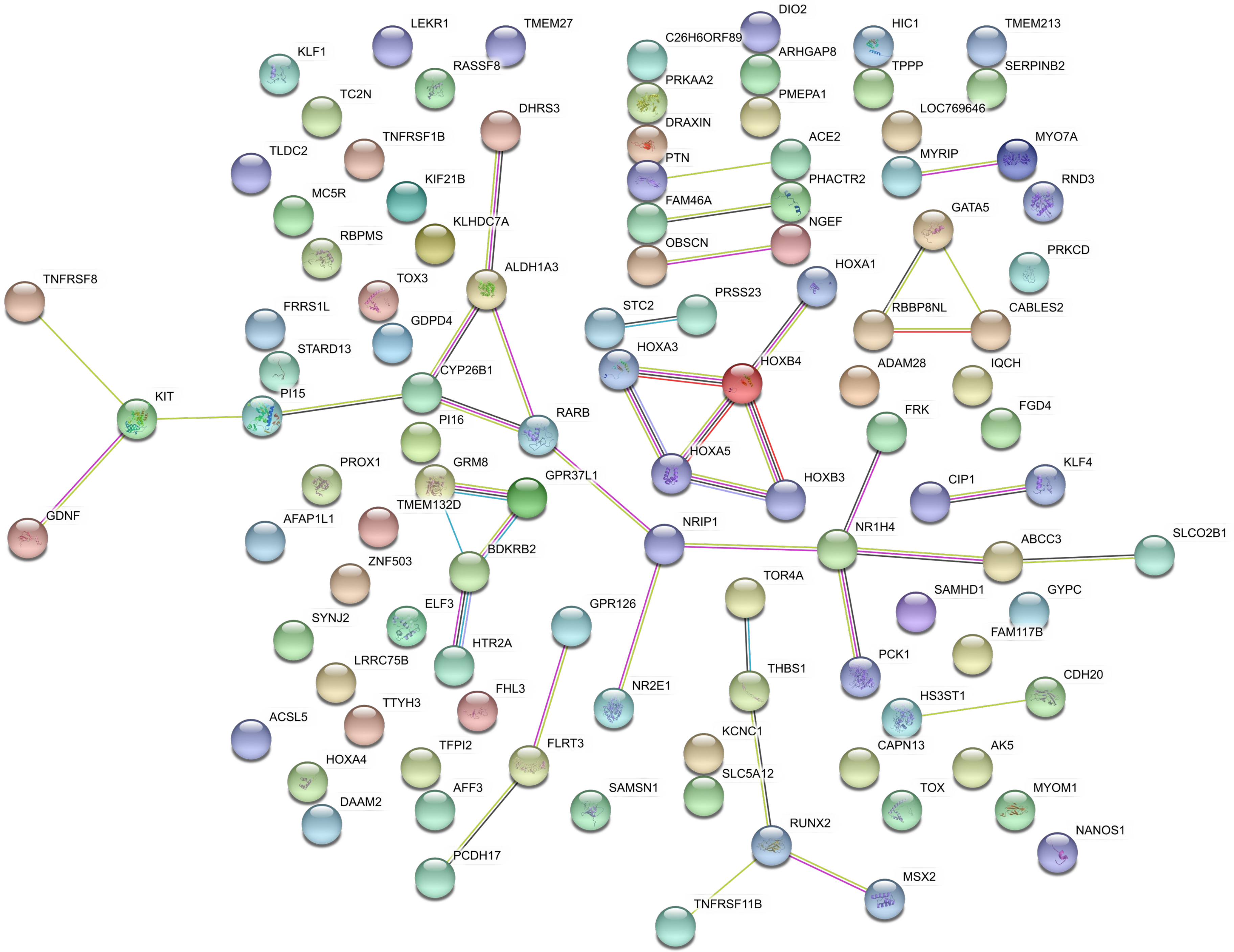

Additional file 9B: Protein interaction network analysis of genes that were differentially expressed in LMH cells after 4h exposure to retinol.

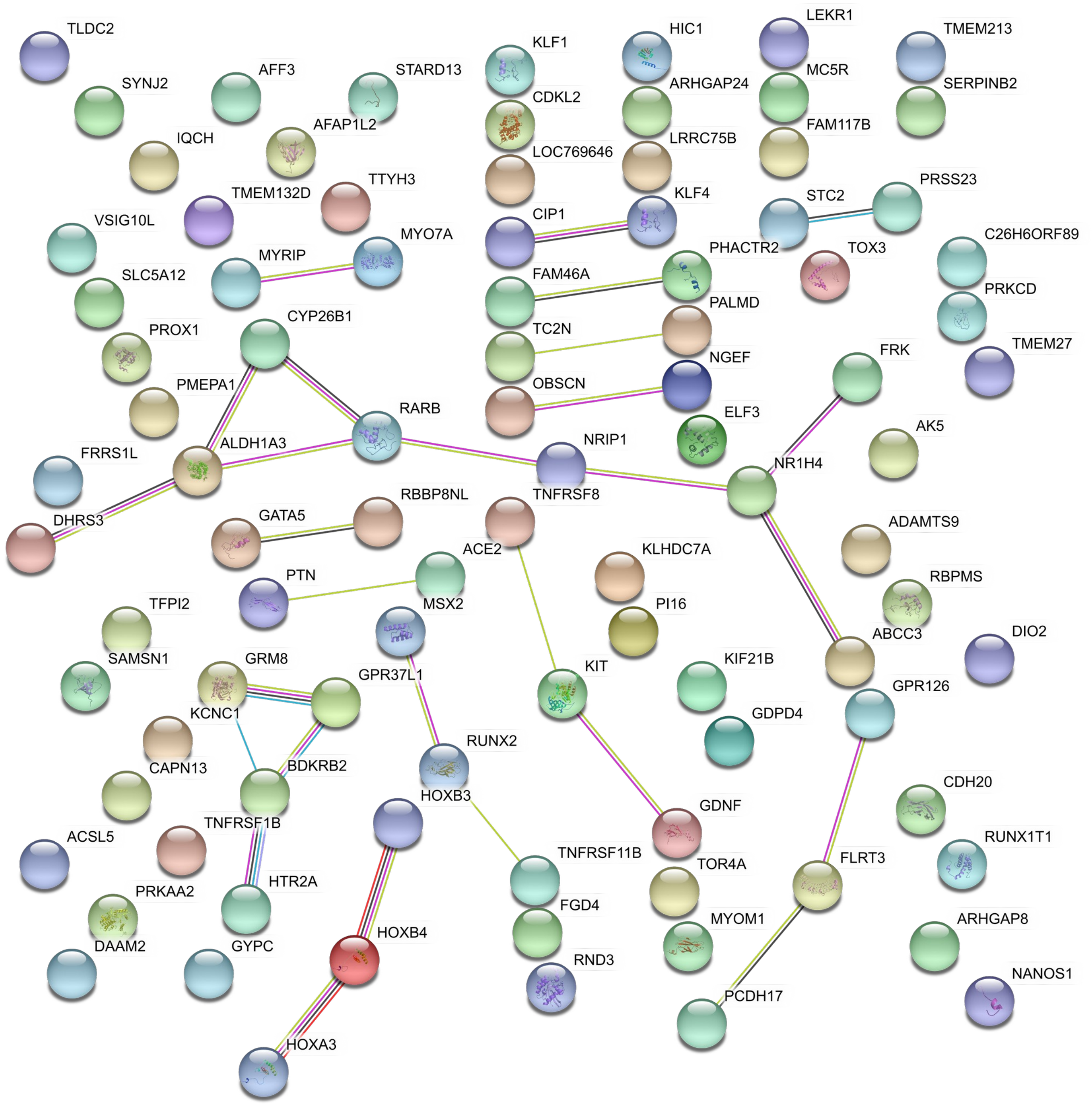

Supplement: Supplementary file 9 — Additional file 9. Protein interaction network analysis results of genes that were differentially expressed in LMH cells after 4 h exposure to retinoic acid or retinol. [file 12864_2021_7451_MOESM9_ESM.pdf]
